# Efficacy of microbicidal actives and formulations for inactivation of Lassa virus in suspension

**DOI:** 10.1038/s41598-023-38954-5

**Published:** 2023-08-10

**Authors:** Todd A. Cutts, Raymond W. Nims, Joseph R. Rubino, Julie McKinney, Jens H. Kuhn, M. Khalid Ijaz

**Affiliations:** 1 Applied Biosafety Research Program, Public Health Agency of Canada, 1015 Arlington St., Winnipeg, MB R3E 3P6 Canada; 2 Syner-G Biopharma, 6000 Spine Road, Suite 201, Boulder, CO 80301 USA; 3 Global Research and Development for Lysol and Dettol, Reckitt Benckiser LLC, One Philips Parkway, Montvale, NJ 07645 USA; 4 Integrated Research Facility at Fort Detrick, National Institute of Allergy and Infectious Diseases, National Institutes of Health, B-8200 Research Plaza, Fort Detrick, Frederick, MD 21702 USA

**Keywords:** Antimicrobials, Pathogens, Virology

## Abstract

The World Health Organization’s R&D Blueprint list of priority diseases for 2022 includes Lassa fever, signifying the need for research and development in emergency contexts. This disease is caused by the arenavirus Lassa virus (LASV). Being an enveloped virus, LASV should be susceptible to a variety of microbicidal actives, although empirical data to support this expectation are needed. We evaluated the virucidal efficacy of sodium hypochlorite, ethanol, a formulated dual quaternary ammonium compound, an accelerated hydrogen peroxide formulation, and a *p*-chloro-*m*-xylenol formulation, per ASTM E1052-20, against LASV engineered to express green fluorescent protein (GFP). A 10-μL volume of virus in tripartite soil (bovine serum albumin, tryptone, and mucin) was combined with 50 μL of disinfectant in suspension for 0.5, 1, 5, or 10 min at 20–25 °C. Neutralized test mixtures were quantified by GFP expression to determine log_10_ reduction. Remaining material was passaged on Vero cells to confirm absence of residual infectious virus. Input virus titers of 6.6–8.0 log_10_ per assay were completely inactivated by each disinfectant within 1–5 min contact time. The rapid and substantial inactivation of LASV suggests the utility of these microbicides for mitigating spread of infectious virus during Lassa fever outbreaks.

## Introduction

The World Health Organization (WHO)’s R&D Blueprint list of priority diseases^1^ comprises a list of diseases for which “… given their potential to cause a public health emergency and the absence of efficacious drugs and/or vaccines, there is an urgent need for accelerated research and development…”^1^. One of the listed diseases is Lassa fever, which is caused by Lassa virus (LASV), an enveloped virus from the *Arenaviridae* family (genus *Mammarenavirus*). This rapidly expanding RNA virus family has been established for a number of genetically related viruses, including several that, like LASV, frequently cause lethal zoonoses. These include Chapare virus, Lujo virus, Machupo virus, Junín virus, Guanarito virus, and Sabiá virus^2^.

One of the concerning knowledge gaps for the diseases highlighted in the WHO R&D Blueprint list of priority diseases, including Lassa fever, is the lack of empirical data for the effectiveness of virucides against the infectious agents that might be used for applications, including air sanitization, skin sanitization, liquid inactivation, and inanimate surface hygiene^3^. The few manuscripts that have been published on the inactivation of LASV by microbicides (acetic acid, formalin, β-propiolactone, and phenol/guanidine thiocyanate) addressed only the efficacy in rendering laboratory specimens safe for handling within diagnostic and histology laboratories. In addition, review of this literature^3^ has indicated that the secondary literature sources for efficacy of more commonly used surface-hygiene microbicides for inactivating arenaviruses, including LASV, have not supported efficacy claims with primary literature citations.

Resolving this particular knowledge gap for LASV and many of the other viruses causing priority list diseases has been limited, in part, due to the need for manipulating such viruses within maximum containment (termed in Northern America biosafety level 4 [BSL-4]) laboratories. In the present study, we were able to leverage the BSL-4 laboratory at Public Health Agency of Canada, Winnipeg, MB, Canada, and a LASV engineered^4^ to express green fluorescent protein (GFP). Using standardized methodologies described in ASTM E-1052-20^5^, we have evaluated the virucidal efficacy of commonly used microbicidal actives (sodium hypochlorite and ethanol), and formulations containing a dual quaternary ammonium compound (QAC), accelerated hydrogen peroxide (AHP), and *p*-chloro-*m*-xylenol (PCMX) against LASV in suspension in the presence of a tripartite soil load^6^. Organic soil loads are used as the challenge matrix to model virus inactivation by microbicides in relevant matrices, such as human sputum or blood. Use of hard water as diluent for specific actives was included in the study design, because it is a known antagonist to microbicidal activity and is commonly available in the field^7^.

In addition to the methodologies in ASTM E-1052-20, our study was made more stringent by decreasing the volume of the microbicides used during testing and by ruling out the presence of residual infectious LASV in the post-disinfection/post-neutralization samples through use of a safety test (performed in addition to the standard quantification of titer reduction). This safety test involved inoculation of 650 µL of undiluted neutralized test sample onto six-well plates containing *Chlorocebus aethiops* kidney epithelial (Vero) cells and passaging any cultures found to be negative for GFP at least twice. This was done to evaluate the virucidal efficacy test for the possibility of any residual virus being present at levels lower than the limit of detection of the 50% tissue culture infectious dose (TCID_50_) titration assay performed in Vero cells per the ASTM standard.

## Methods

### Cell line, virus, and culture medium

Grivet (*Chlorocebus aethiops*) kidney epithelial (Vero) cells (American Type Culture Collection [ATCC], Manassas, VA, USA; #CCL-81) were maintained at 37 °C with 5% carbon dioxide (CO_2_) in minimum essential medium (MEM; HyClone, Logan, UT, USA) supplemented with 5% heat-inactivated fetal calf serum (FCS; Gibco, Grand Island, NY, USA) and 10 units per mL penicillin/streptomycin (pen/strep; Gibco). Lassa virus (Josiah strain) expressing green fluorescent protein (LASV-GFP) was amplified and used, as previously described^4^, for all assays. All culture manipulations involving LASV-GFP were performed in a BSL-4 laboratory at the Canadian Science Centre for Human and Animal Health, Winnipeg, MB, Canada.

### Stock virus preparation

A characterized stock of LASV-GFP was prepared by exposing ten T75 flasks of Vero cells at ≈80% confluency at a multiplicity of infection of 0.01 viral particles per cell. At 4 d post-infection, essentially all cells within the confluent cell monolayers were observed to express GFP. The flasks were then placed in a freezer at − 70 °C. The frozen flasks were thawed on the following day and the conditioned media was removed and clarified by low-speed centrifugation (4500 × *g*) for 10 min. The resulting supernatants were pooled and layered onto 20% w/v sucrose cushions prepared in Tris-NaCl-EDTA buffer prepared in house. The pooled supernatants were subjected to centrifugation (≈130,000 × *g* for 2 h), and the viral pellets obtained were resuspended in MEM containing 2% FCS and 10 units per mL pen/strep to create virus culture medium (VCM). The virus pool obtained was aliquoted into small portions that were frozen at − 70 °C. The stock virus titer was found to be > 9.2 log_10_ per mL, as determined by TCID_50_ assay, using the method described by Reed and Muench^8^.

### Microbicides

A variety of microbicidal actives or formulations containing microbicidal actives were evaluated for virucidal efficacy against LASV-GFP. The microbicides tested are listed in Table 1, along with sources and the supplied and tested concentrations.

**Table 1:** Microbicides/microbicidal formulations and concentrations as supplied and as evaluated.

Microbicide	Active ingredient	Formulation	Source (part number)	Concentration supplied (%)	In test concentration (dilution of the supplied reagent)
Bleach	Sodium hypochlorite (NaOCl)	No	Imperial Soap and Supplies, LTD. (IMP750-1)	12	0.5% [5000 ppm] (1:24)
Ethanol	Ethanol (EtOH)	No	Commercial Alcohols, Inc. (P016EA95)	95	67% (2.39:1)
MicroChem plus	Dual quaternary ammonium compound (dual QAC)	Yes	National Chemical Laboratories, Inc. (0255)	100	2% (1:50)
PreEmpt	Accelerated hydrogen peroxide (AHP)	Yes	Contec (11,305)	100	2.5% (1:40)
Dettol	*p*-chloro-*m*-xylenol (PCMX)	Yes	Reckitt (29 636 6215)	4.8	0.12% (1:40)
					0.06% (1:80)
					0.04% (1:120)

The unformulated microbicidal actives and the formulations tested were prepared in hard water^7^ (prepared as 1 L deionized water supplemented with 0.4 g calcium carbonate) on the day of assay. The resulting solutions were inverted to mix and used within 2.5 to 4 h of preparation.

### Assessment of microbicide neutralization by chemical reagents and Amicon columns

Neutralizing reagents or mechanical removal using Amicon filter columns were evaluated for ability to neutralize the virucidal effects of the microbicides to enable investigation of specific contact times, and/or to mitigate the cytotoxic effects of the microbicides on the Vero cells used to assay residual virus. The procedures used are described in Supplemental Materials.

### Microbicide virucidal efficacy testing

**Figure 1:**
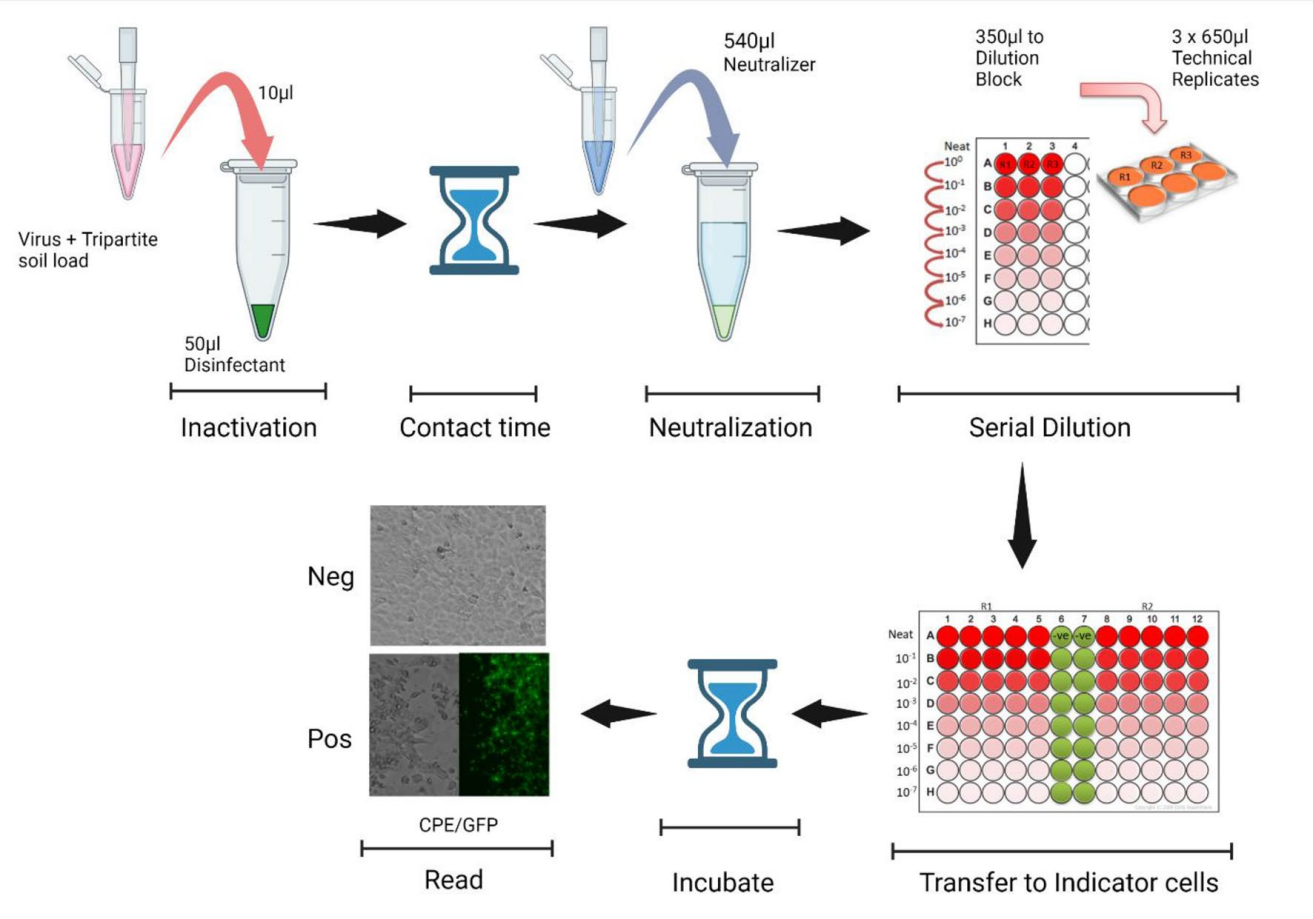
Schematic representation of the suspension inactivation efficacy testing methodology performed using neutralization by VCM (minimal essential medium + 2% fetal calf serum + 10 units per mL penicillin/streptomycin) alone. The entire procedure was performed three times for each microbicide evaluated, in three technical replicates each, as depicted.

**Figure 2:**
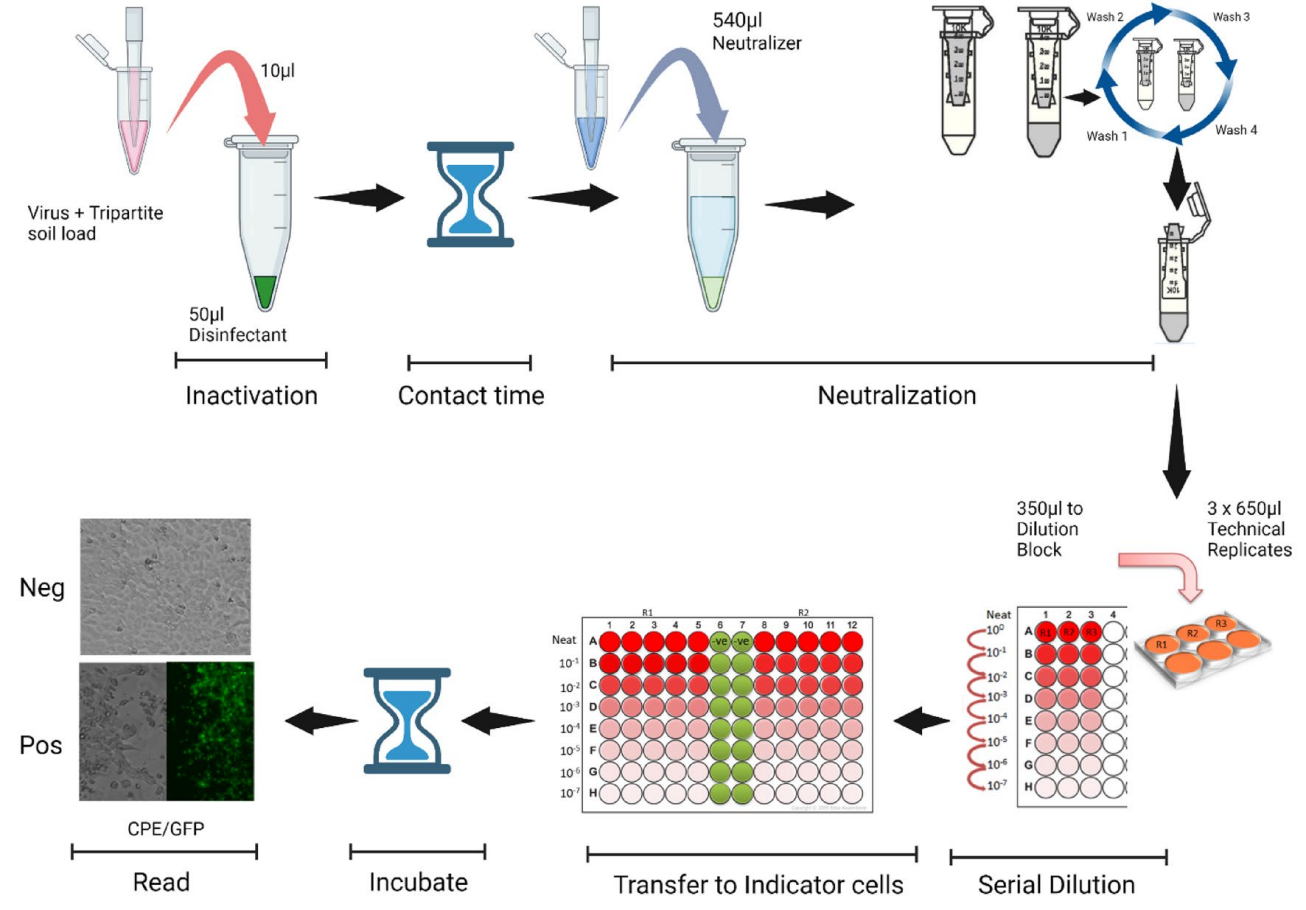
Schematic representation of the suspension inactivation efficacy testing methodology using VCM (minimal essential medium + 2% fetal calf serum + 10 units per mL penicillin/streptomycin) and mechanical neutralization using Amicon Spin Columns. The entire procedure was performed three times for each microbicide evaluated, in three technical replicates each, as depicted.

The LASV-GFP suspension inactivation efficacy testing of the microbicides (Fig. 1) was conducted at ambient temperature using ASTM E-1052-20^5^. A modification to the standard method was made to reduce the volume of test microbicide, increasing the stringency of the evaluation. Stocks of LASV-GFP in tripartite soil load^6^ were prepared on the day of assay. Briefly, a single tube of stock virus was removed from frozen storage and mixed with a tripartite soil load (≈1.7 × 10^9^ TCID_50_ per mL virus, 0.25% bovine serum albumin, 0.35% tryptone, 0.08% mucin). The virus/tripartite soil load mixture (10 µL) was added to 50 µL of prepared microbicide or to 50 µL of VCM to create the positive virus control. A similar preparation was used to challenge the efficacy of the test microbicides. The virus/tripartite soil mixture was incubated with the test microbicide at room temperature for contact times of 30 s, 1 min, 5 min, and 10 min. At the end of each time point, the microbicides were neutralized, either by adding 940 µL of VCM (Fig. 1) or by adding 500 μL VCM and filtering out the microbicides using a YM100 Amicon column (Fig. 2). All studies involving LASV-GFP were conducted within a BSL-4 facility.

### Mechanical neutralization using Amicon filter columns

For microbicides that could not be adequately neutralized using VCM alone (AHP, PCMX, and dual QAC), a mechanical filtration procedure via Amicon YM100 columns (UFCS510096; EMD Millipore, Darmstadt, Germany) was used during virucidal efficacy testing (Fig. 2). After the planned contact times, virus-microbicide suspensions were diluted with 500 μL of VCM and immediately eluted through the columns. In accordance with the manufacturer’s procedures, the columns were centrifuged for 3 min at 14,000 × *g* and the flow-through was discarded. To the retentate, 400 μL of fresh VCM were then added to the filter cup, centrifuged for an additional 3 min at 14,000 × *g*, and the flow-through was again discarded. To elute retained virus from the column, 500 μL of fresh VCM were added to the filter cup, incubated for 2 min, inverted into a fresh tube, and spun for 2 min at 1000 × *g*. The final eluted volumes were brought to 1000 μL with VCM for evaluation. A single wash step was performed for the AHP and PCMX, whereas four wash steps were needed for the dual QAC.

In either case (neutralization using VCM alone or VCM plus neutralization using Amicon columns), a 350-µL portion of neutralized test solution was assayed for residual infectious virus titer using a ten-fold dilution scheme in VCM, with 50 µL of each resulting dilution being added to 96-well plates of Vero cells (n = 5 replicate wells per dilution). The inoculated cell monolayers were scored 5 d post-infection for GFP, and virus titers (in units of TCID_50_) were calculated according to the Reed-Muench method^8^.

### Plate safety test

In addition to the 96-well plate TCID_50_ assay described above, neutralized material also was evaluated for low levels of infectious virus in a plate safety test. In this test, which is used when dealing with especially lethal challenge viruses, 650 μL of remaining undiluted neutralized test sample (one sample for each technical replicate) were added to Vero cells in wells of a six-well plate containing 4 mL of VCM. In addition, an inoculum containing < 5 TCID_50_ units of LASV-GFP was used in each six-well plate to serve as a positive control. All wells were scored 5 d post-infection for presence or absence of the fluorescence associated with GFP (indicative of LASV-GFP-infected cells). The confirmed ability of this assay to detect very low concentrations (< 5 TCID_50_ units) of the positive control virus indicates its sensitivity and provides confidence that a negative result in the separate TCID_50_ assay truly reflects the absence of residual infectious LASV-GFP in the neutralized test samples.

### Analysis of viral inactivation efficacy

For each disinfectant, three separate independent assays were conducted with each time point having three technical replicates within each assay. TCID_50_ titers for positive virus controls and neutralized microbicide test conditions were determined using the method of Reed and Muench^8^. The log_10_ reduction values achieved by the microbicides at given contact times were calculated by subtracting the post-disinfection log_10_ TCID_50_ values (titers) from the log_10_ TCID_50_ values obtained for the corresponding positive virus control. Statistical comparison of the mean (n = 5 replicates) viral titers obtained in the neutralization effectiveness studies (Supplemental Fig. S1) was performed using a non-parametric unpaired t-test, with statistical significance set at *p* < 0.05.

## Results

### Neutralization effectiveness evaluation

The results from the determination of the effectiveness of the neutralization procedures (chemical or mechanical) are provided in the Supplemental Materials. It was found that 0.5% sodium hypochlorite and 67% ethanol could be adequately neutralized using VCM alone. VCM plus mechanical neutralization using Amicon columns was required for dual QAC, AHP, and PCMX.

### Virucidal efficacy of 0.5% sodium hypochlorite for LASV

**Figure 3:**
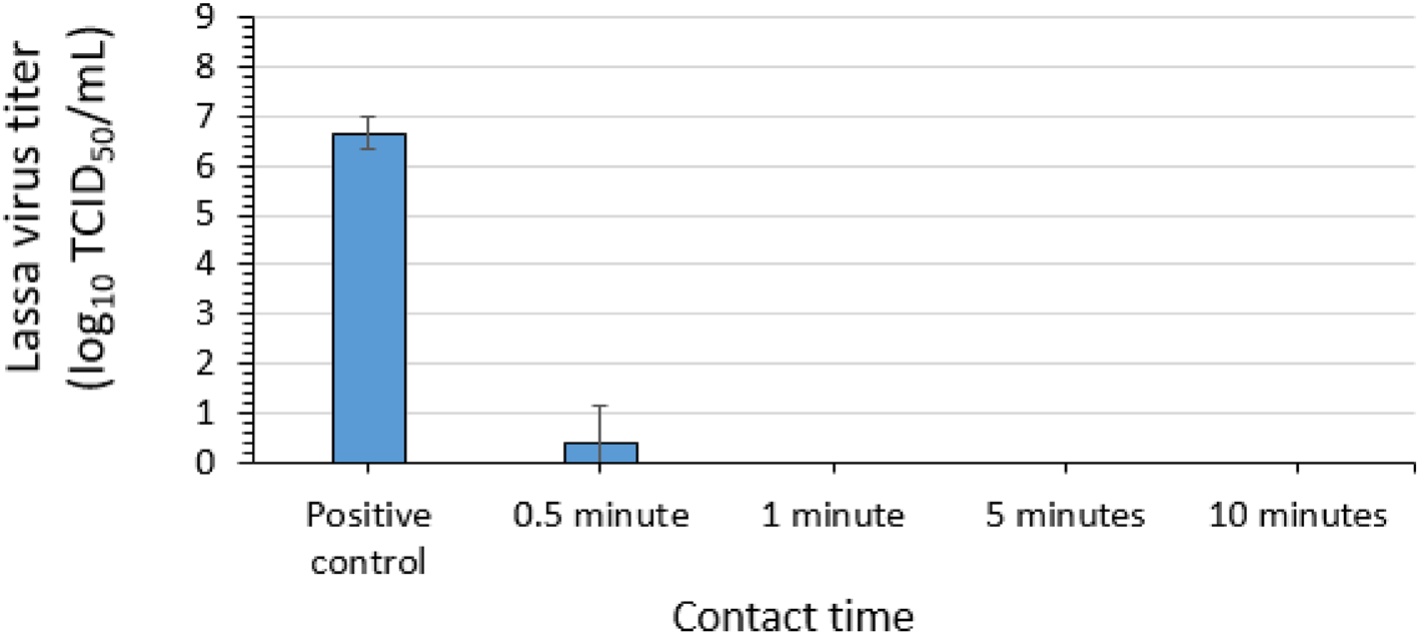
Efficacy of 0.5% sodium hypochlorite for inactivating Lassa virus in suspension. Error bars indicate standard deviation of the mean for n = 3 independent studies with 3 technical replicates each.

Three replicate evaluations of the efficacy of 0.5% (5000 ppm) sodium hypochlorite (NaOCl) (Table 1) for inactivating LASV-GFP virus in suspension were conducted. Contact times of 0.5, 1, 5, and 10 min were evaluated at ambient temperature. A mean LASV-GFP titer of 6.66 log_10_ TCID_50_ per mL (4.6 × 10^6^ TCID_50_ per mL) was recovered for the positive control (no disinfectant) (Fig. 3). The post-exposure/neutralization titer for the 0.5% sodium hypochlorite condition was 0.42 log_10_ TCID_50_ per mL after a 0.5-min (30-s) contact time, representing a 6.2 log_10_ reduction. After 1, 5, and 10 min of contact with 0.5% sodium hypochlorite, complete inactivation (≥ 6.7 log_10_) of LASV-GFP was observed (Fig. 3).

Further evidence of the complete inactivation of LASV-GFP following the 1-, 5-, and 10-min contact times was obtained in the plate safety test (Table 2). In this assay (depicted schematically in Fig. 2), undiluted post-exposure/neutralization mixture (650 μL per technical replicate onto one well per replicate) was added to six-well plates of Vero cells, which were passaged up to two times to determine residual infectious virus. One technical replicate from one assay of the 0.5-min contact time displayed GFP in this assay. No evidence of residual infectious virus was obtained from the technical replicates for the 1-, 5-, and 10-min contact times (Table 2).

**Table 2:** Safety plate test for inactivation of Lassa virus by 0.5% sodium hypochlorite in suspension.

	Assay 1			Assay 2			Assay 3		
	GFP (+/−)	GFP (+/−)	GFP (+/−)	GFP (+/−)	GFP (+/−)	GFP (+/−)	GFP (+/−)	GFP (+/−)	GFP (+/−)
	10^0^ Rep 1	10^0^ Rep 2	10^0^ Rep 3	10^0^ Rep 1	10^0^ Rep 2	10^0^ Rep 3	10^0^ Rep 1	10^0^ Rep 2	10^0^ Rep 3
0.5 min	−	−	+	−	−	−	−	−	−
1 min	−	−	−	−	−	−	−	−	−
5 min	−	−	−	−	−	−	−	−	−
10 min	−	−	−	−	−	−	−	−	−

GFP, green fluorescent protein; + , GFP detected; −, GFP not detected.

### Virucidal efficacy of 67% ethanol for LASV

**Figure 4:**
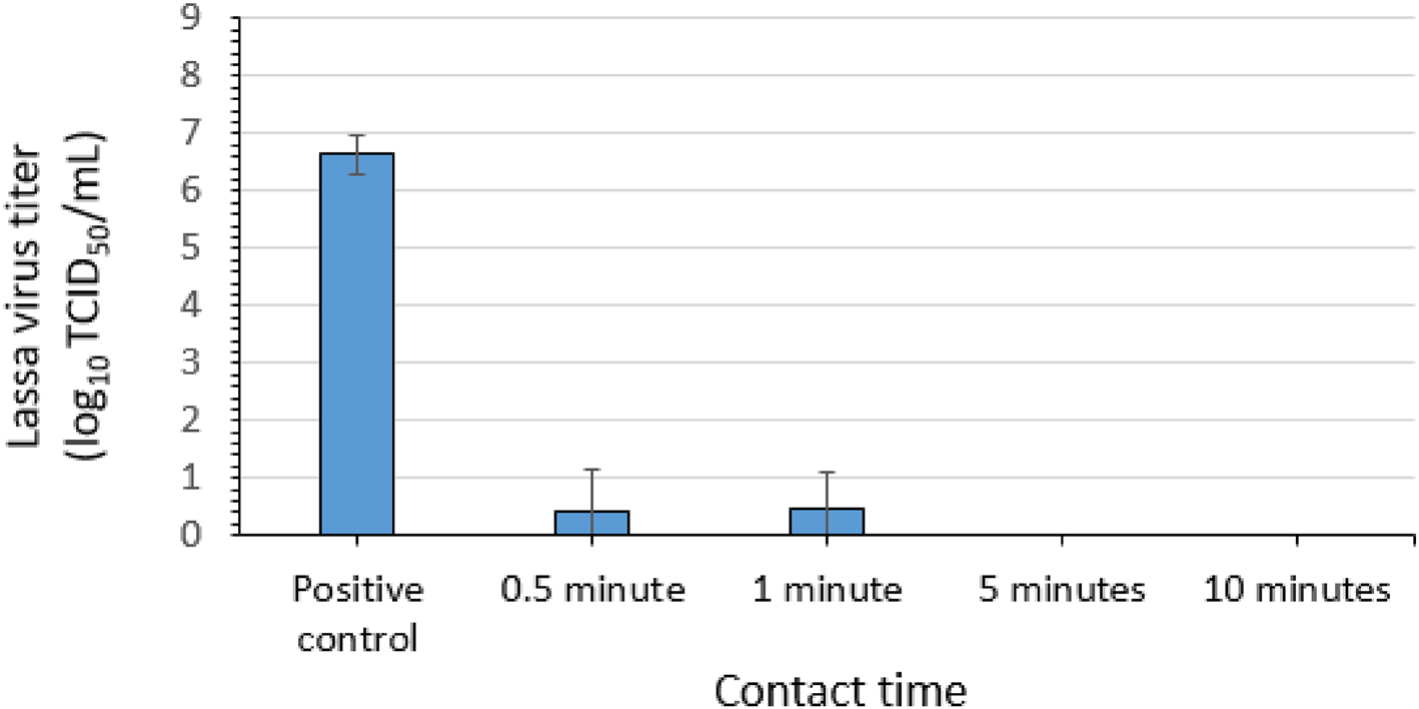
Efficacy of 67% ethanol for inactivating Lassa virus in suspension. Error bars indicate standard deviation of the mean for n = 3 independent studies with 3 technical replicates each.

Three replicate evaluations of the efficacy of 67% ethanol (EtOH) (Table 1) for inactivating LASV-GFP virus in suspension were conducted. Contact times of 0.5, 1, 5, and 10 min were evaluated at ambient temperature. A mean LASV-GFP titer of 6.62 log_10_ TCID_50_ per mL (4.4 × 10^6^ TCID_50_ per mL) was recovered for the positive control (no disinfectant) (Fig. 4). The post-exposure/neutralization titers for the 67% ethanol condition were 0.42 log_10_ TCID_50_ per mL for the 0.5-min (30-s) contact time and 0.42 log_10_ TCID_50_ per mL for the 1-min contact time. These correspond to reductions of 6.2 log_10_. After 5 and 10 min of contact with 67% ethanol, complete inactivation (≥ 6.6 log_10_) of LASV-GFP was observed (Fig. 4).

Further evidence of the complete inactivation of LASV-GFP following the 5- and 10-min contact times was obtained in the plate safety test. One technical replicate from one assay of the 0.5 min contact time and three technical replicates from two assays of the 1-min contact time displayed GFP in this assay. No evidence of residual infectious virus was obtained from the technical replicates for the 5- and 10-min contact times (Table 3).

**Table 3:** Plate safety test for inactivation of Lassa virus by 67% ethanol in suspension. GFP, green fluorescent protein.

	Assay 1			Assay 2			Assay 3		
	GFP (+/−)	GFP (+/−)	GFP (+/−)	GFP (+/−)	GFP (+/−)	GFP (+/−)	GFP (+/−)	GFP (+/−)	GFP (+/−)
	10^0^ Rep 1	10^0^ Rep 2	10^0^ Rep 3	10^0^ Rep 1	10^0^ Rep 2	10^0^ Rep 3	10^0^ Rep 1	10^0^ Rep 2	10^0^ Rep 3
0.5 min	−	−	−	−	−	−	−	−	+
1 min	+	−	−	−	−	−	+	+	−
5 min	−	−	−	−	−	−	−	−	−
10 min	−	−	−	−	−	−	−	−	−

GFP, green fluorescent protein; + , GFP detected; −, GFP not detected.

### Virucidal efficacy of a dual QAC formulation for LASV

**Figure 5:**
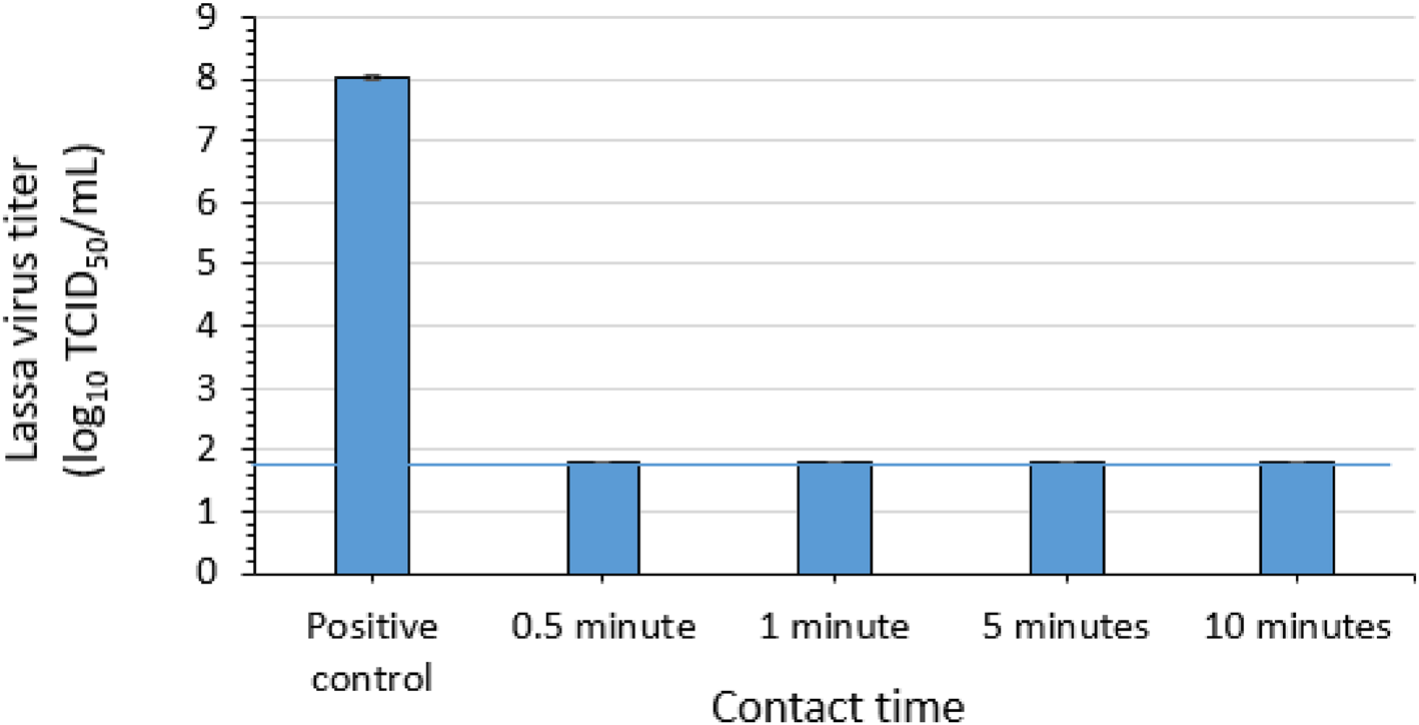
Efficacy of a 2% dual quaternary ammonium compound (QAC) formulation for inactivating Lassa virus in suspension. The limit of detection of the titration assay (1.8 × 10^1^ TCID_50_ per mL) is indicated by the solid blue horizontal line. Error bars indicate standard deviation of the mean for n = 3 independent studies with 3 technical replicates each.

Three replicate evaluations of the efficacy of a dual QAC formulation (Table 1) for inactivating LASV-GFP virus in suspension were conducted. Contact times of 0.5, 1, 5, and 10 min were evaluated at ambient temperature. A mean LASV-GFP titer of 8 log_10_ TCID_50_ per mL was recovered for the positive control (no disinfectant) (Fig. 5). The post-exposure/neutralization titers for the dual QAC conditions were ≤ 1.8 log_10_ TCID_50_ per mL (the defined limit of detection of the titration assay) for all contact times (Fig. 5). The assay limit of detection was determined by residual cytotoxicity to the Vero cells of the neutralization mixture following elution from the Amicon column. These results indicate a reduction in titer of LASV of ≤ 6.2 log_10_ for each contact time.

In the case of the dual QAC formulation, the plate safety test was not able to be conducted, due to the residual cytotoxicity of the undiluted post-exposure/neutralization samples to the Vero cells despite repeated mitigation efforts using multiple filtrations via Amicon columns.

### Virucidal efficacy of an AHP formulation for LASV

**Figure 6:**
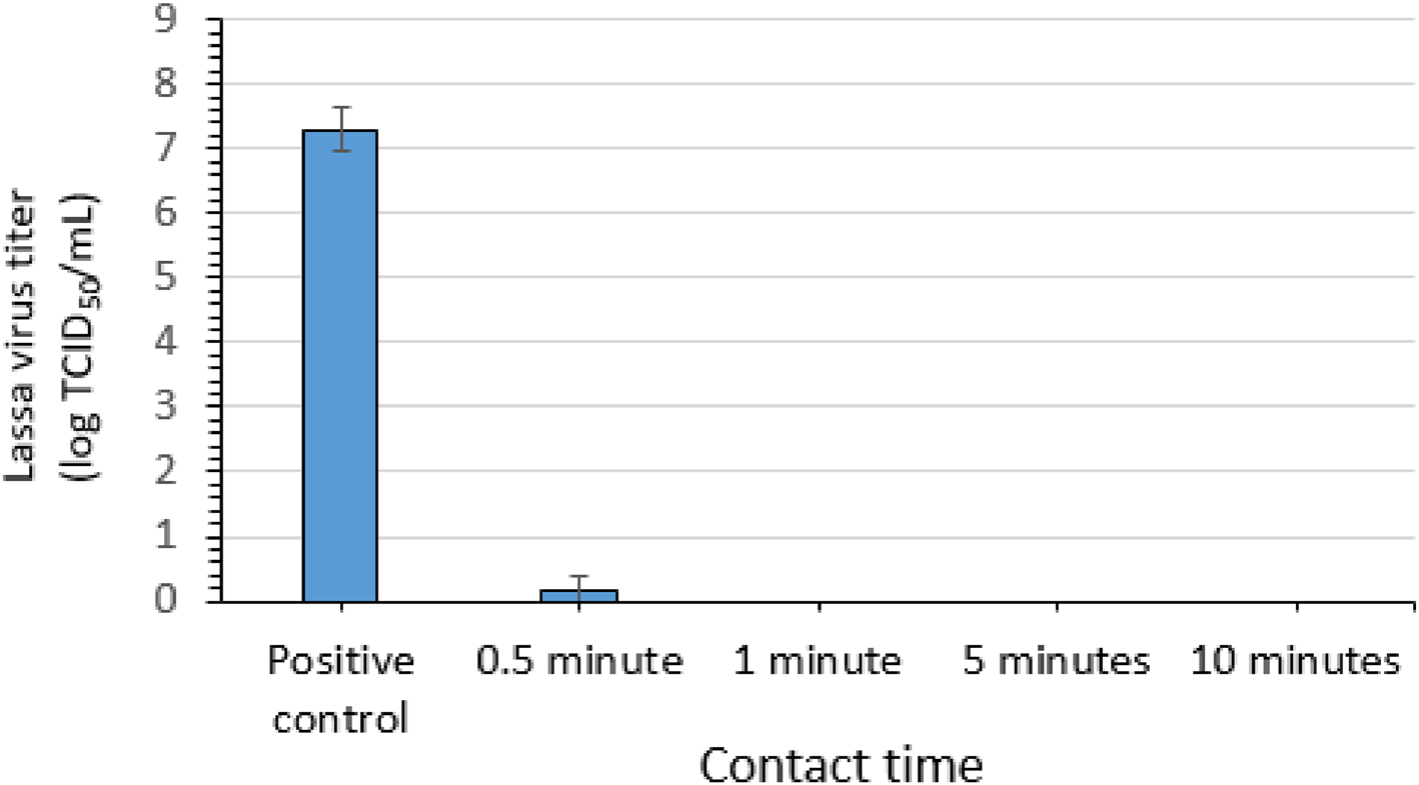
Efficacy of an accelerated hydrogen peroxide formulation (AHP; 1:40 dilution) for inactivating Lassa virus in suspension. Error bars indicate standard deviation of the mean for n = 3 independent studies with 3 technical replicates each.

Three replicate evaluations of the efficacy of an AHP formulation (Table 1) for inactivating LASV-GFP virus in suspension were conducted. Contact times of 0.5, 1, 5, and 10 min were evaluated at ambient temperature. A mean LASV-GFP titer of 7.3 log_10_ TCID_50_ per mL was recovered for the positive control (no disinfectant) (Fig. 6). The post-exposure/neutralization titer for the AHP condition was 0.17 log_10_ TCID_50_ per mL for the 0.5-min (30-s) contact time, representing a 7.1 log_10_ reduction. After 1, 5, and 10 min of contact with AHP, complete inactivation (≥ 7.3 log_10_) of LASV-GFP was observed (Fig. 6).

Further evidence of the complete inactivation of LASV-GFP following the 1-, 5-, and 10-min contact times for AHP was obtained in the plate safety test. Two technical replicates from one assay of the 0.5-min (30-s) contact time displayed GFP in this assay. No evidence of residual infectious virus was obtained from the technical replicates for the 1-, 5-, 10-min contact times (Table 4).

**Table 4:** Plate safety test for inactivation of Lassa virus by an accelerated hydrogen peroxide formulation (AHP; 1:40 dilution) in suspension.

	Assay 1			Assay 2			Assay 3		
	GFP (+/−)	GFP (+/−)	GFP (+/−)	GFP (+/−)	GFP (+/−)	GFP (+/−)	GFP (+/−)	GFP (+/−)	GFP (+/−)
	10^0^ Rep 1	10^0^ Rep 2	10^0^ Rep 3	10^0^ Rep 1	10^0^ Rep 2	10^0^ Rep 3	10^0^ Rep 1	10^0^ Rep 2	10^0^ Rep 3
0.5 min	−	+	+	−	−	−	−	−	−
1 min	−	−	−	−	−	−	−	−	−
5 Minutes	−	−	−	−	−	−	−	−	−
10 Minutes	−	−	−	−	−	−	−	−	−

GFP, green fluorescent protein; + , GFP detected; −GFP not detected.

### Virucidal efficacy of a PCMX formulation for LASV

**Figure 7:**
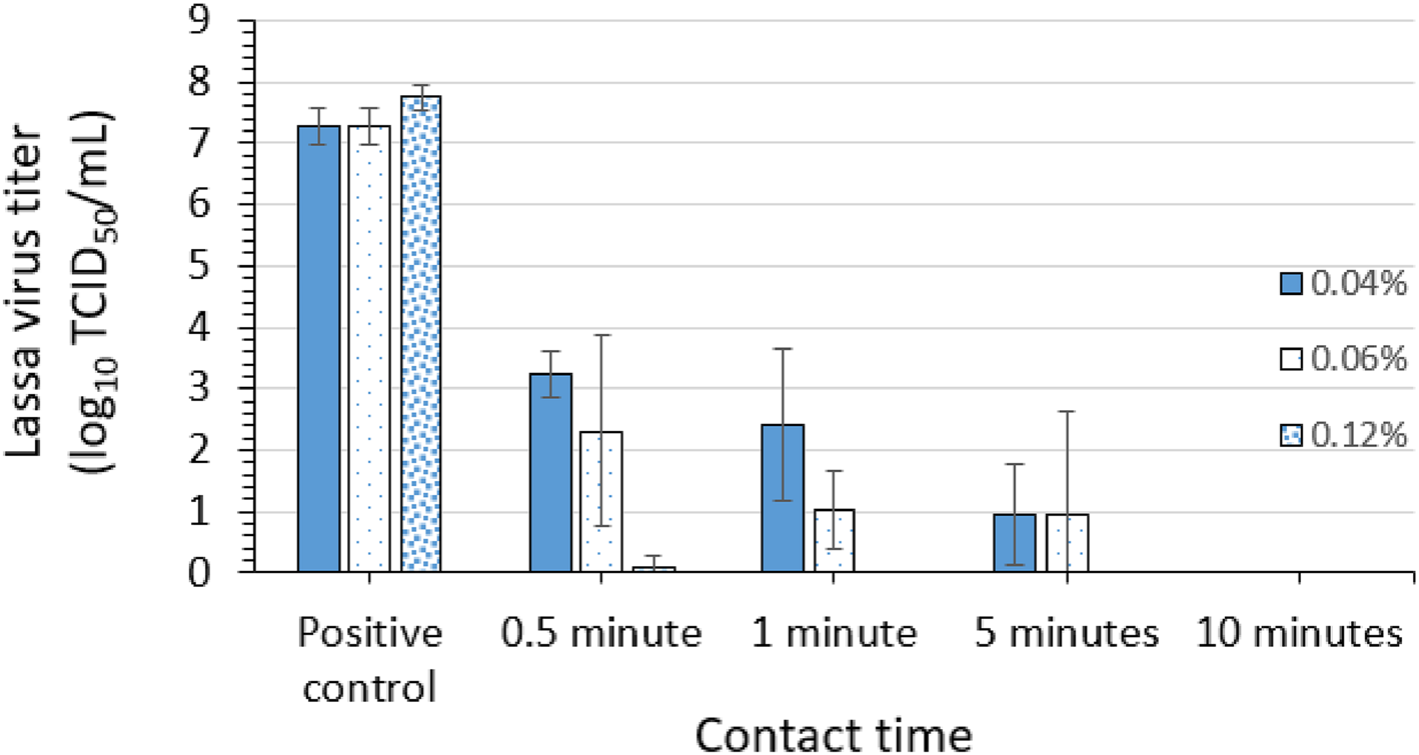
Efficacy of *p*-chloro-*m*-xylenol (PCMX) at test concentrations of 0.04, 0.06, and 0.12% for inactivating Lassa virus in suspension. Error bars indicate standard deviation of the mean for n = 3 independent studies with 3 technical replicates each.

Three replicate evaluations of the efficacy of three in-test concentrations (0.04, 0.06, and 0.12%) of PCMX in a commercial formulation (Table 1) for inactivating LASV-GFP virus in suspension were conducted. Contact times of 0.5, 1, 5, and 10 min were evaluated at ambient temperature. Mean LASV-GFP titers of 7.8 log_10_ TCID_50_ per mL, 7.3 log_10_ TCID_50_ per mL, and 7.3 log_10_ TCID_50_ per mL were recovered for the positive control (no disinfectant) conditions for the assay of the 0.12, 0.06, and 0.04% in-test concentrations of PCMX, respectively (Fig. 7).

PCMX concentration-dependent inactivation of LASV-GFP was observed at the various contact times. For instance, the post-exposure/neutralization titers for the 0.12, 0.06, and 0.04% PCMX conditions were 0.9 log_10_ TCID_50_ per mL, 2.3 log_10_ TCID_50_ per mL, and 3.2 log_10_ TCID_50_ per mL, respectively, after 0.5-min (30-s) contact time, representing log_10_ reductions of 6.9, 5.0, and 4.1 log_10_, respectively. Following a 1-min contact time, the 0.12% PCMX condition had no viable virus remaining post-exposure/neutralization, whereas for the 0.06 and 0.04% PCMX conditions 1.0 and 2.4 log_10_ TCID_50_ per mL, respectively, were recovered representing log_10_ reductions of 6.3 and 4.9 log_10_, respectively. Following a 5-min contact time, the post-exposure/neutralization titers for the 0.06 and 0.04% PCMX conditions were 0.97 and 0.94 log_10_ TCID_50_ per mL, respectively, representing log_10_ reductions of 6.3 and 6.4 log_10_, respectively. Complete inactivation (≥ 7.8 log_10_) of LASV-GFP was afforded by 0.12% PCMX at contact times of 1, 5, and 10 min. For the 0.06 and 0.04% PCMX concentrations, complete inactivation (≥ 7.3 log_10_) of LASV-GFP was observed at the 10-min contact time (Fig. 7).

Further evidence of the complete inactivation of LASV-GFP following the 1-, 5-, and 10-min contact times for 0.12% PCMX, and following the 10-min contact times for 0.06 and 0.04% PCMX, was obtained in the plate safety tests (Tables 5, 6, 7). One technical replicate from one assay of the 0.5-min contact time displayed GFP in the plate safety test for 0.12% PCMX, whereas no evidence of residual infectious virus was obtained from the technical replicates for the 1-, 5-, and 10-min contact times (Table 5).

**Table 5:** Plate safety test for inactivation of Lassa virus by 0.12% *p*-chloro-*m*-xylenol (PCMX) in suspension.

	Assay 1			Assay 2			Assay 3		
	GFP (+/−)	GFP (+/−)	GFP (+/−)	GFP (+/−)	GFP (+/−)	GFP (+/−)	GFP (+/−)	GFP (+/−)	GFP (+/−)
	10^0^ Rep 1	10^0^ Rep 2	10^0^ Rep 3	10^0^ Rep 1	10^0^ Rep 2	10^0^ Rep 3	10^0^ Rep 1	10^0^ Rep 2	10^0^ Rep 3
0.5 min	−	−	−	−	−	−	−	−	+
1 min	−	−	−	−	−	−	−	−	−
5 min	−	−	−	−	−	−	−	−	−
10 min	−	−	−	−	−	−	−	−	−

GFP, green fluorescent protein; + , GFP detected; −, GFP not detected.

As expected on the basis of the titration assay results, multiple replicates for one or more individual assays for the 0.06 and 0.04% PCMX concentrations demonstrated GFP following the 0.5-, 1-, and 5-min contact times, confirming the presence of residual infectious virus in these replicates. No evidence of residual infectious virus was obtained from the technical replicates for the 10-min contact times for the 0.06 and 0.04% PCMX exposures (Tables 6 and 7).

**Table 6:** Plate safety test for inactivation of Lassa virus by 0.06% *p*-chloro-*m*-xylenol (PCMX) in suspension.

	Assay 1			Assay 2			Assay 3		
	GFP (+/−)	GFP (+/−)	GFP (+/−)	GFP (+/−)	GFP (+/−)	GFP (+/−)	GFP (+/−)	GFP (+/−)	GFP (+/−)
	10^0^ Rep 1	10^0^ Rep 2	10^0^ Rep 3	10^0^ Rep 1	10^0^ Rep 2	10^0^ Rep 3	10^0^ Rep 1	10^0^ Rep 2	10^0^ Rep 3
0.5 min	+	+	+	+	−	+	+	+	+
1 min	−	−	−	−	−	−	+	+	+
5 min	−	−	−	+	−	−	−	+	−
10 min	−	−	−	−	−	−	−	−	−

GFP, green fluorescent protein; + , GFP detected; −, GFP not detected.

**Table 7:** Plate safety test for inactivation of Lassa virus by 0.04% *p*-chloro-*m*-xylenol (PCMX) in suspension.

	Assay 1			Assay 2			Assay 3		
	GFP (+/−)	GFP (+/−)	GFP (+/−)	GFP (+/−)	GFP (+/−)	GFP (+/−)	GFP (+/−)	GFP (+/−)	GFP (+/−)
	10^0^ Rep 1	10^0^ Rep 2	10^0^ Rep 3	10^0^ Rep 1	10^0^ Rep 2	10^0^ Rep 3	10^0^ Rep 1	10^0^ Rep 2	10^0^ Rep 3
0.5 min	+	+	+	+	+	+	+	+	+
1 min	−	+	+	+	+	+	+	+	+
5 min	+	+	−	−	−	−	−	−	−
10 min	−	−	−	−	−	−	−	−	−

GFP, green fluorescent protein; + , GFP detected; −, GFP not detected.

## Discussion

Per WHO^9^, Lassa fever is endemic in a number of Western African countries, including Benin, Ghana, Guinea, Liberia, Mali, Sierra Leone, and Nigeria. Cases have been reported as recently as March 2023 in Ghana^9^. Natal mastomys rats (murid *Mastomys natalensis*) are the primary reservoirs of the causal virus, LASV. Transmission to humans occurs primarily through contact with urine or feces of infected mastomys, but human-to-human transmission via direct contact with blood or bodily fluids may also occur, especially in hospital settings^9^. These considerations suggest that inanimate surface hygiene and liquid inactivation methods for LASV might limit dissemination of the virus to humans.

Partly because maximum containment is needed for conduct of inactivation studies on LASV, there is little published information on the efficacy of microbicides for inactivation of this arenavirus. A recent review of the available primary data on efficacy of microbicides against LASV^3^ revealed that the limited published data pertained to the inactivation of laboratory specimens intended for diagnostic or histology applications^10–14^. The paucity of virucidal data of microbicides against LASV is also emphasized in a recent review of inactivation of emerging viruses in aqueous phase^15^. Our study was intended to resolve this knowledge gap, supplying efficacy information for commonly used microbicidal actives and formulations applicable to inactivation of LASV in liquid suspension.

The United States Environmental Protection Agency (US EPA) recognizes that microbicidal efficacy data may not be available for newly emerging viruses, especially those requiring BSL-4 laboratories for handling the viruses safely. The EPA, therefore, enacted a policy in 2016 enabling efficacy claims against emerging viruses to be made without having provided registration data specifically for those viruses. In its Guidance to Registrants^16^, the EPA has made note of the hierarchy of pathogen susceptibility to microbicides^17–21^ in recognizing that efficacy against one enveloped virus implies efficacy against other enveloped viruses. The EPA policy provides a “process that can be used to identify effective disinfectant products for use against emerging viral pathogens and to permit registrants to make limited claims of their product’s efficacy against such pathogens.”^16^. The guidance outlines “a voluntary two stage process, involving product label amendments and modified terms of registration and applies only to emerging viruses”^16^.

The EPA policy provides inanimate surface hygiene and liquid inactivation alternatives, helpful for use during virus disease outbreaks. Despite this, obtaining empirical data for specific emerging viruses is required for assurance of efficacy against the more lethal viruses. On the basis of information^3^ derived from testing enveloped viruses (such as, Ebola virus and SARS-CoV-2), lipid-disrupting agents—including ethanol, quaternary ammonium compounds (such as the dual QAC compound evaluated), and phenolics (such as PCMX)—were expected to be effective against other enveloped viruses, such as LASV. Certain microbicidal actives and formulations were considered mechanistically to be protein-denaturing agents (ethanol, PCMX, AHP, and sodium hypochlorite) or genome-degrading agents (ethanol, AHP, and sodium hypochlorite). In fact, our study found that these agents caused rapid (i.e., within 30 s contact time) and highly effective (≥ 6 log_10_) inactivation of LASV-GFP when tested in suspension in a tripartite soil load with hard water as diluent to simulate field use of dilutable products, including PCMX and sodium hypochlorite (Table 1).

The standardized ASTM E-1052-20 methodology^5^ is based on demonstrating a reduction in infectious virus titer after exposure to a test microbicide. These data are then available for making EPA disinfectant efficacy claims. For instance, the EPA stated the following in its 2012 disinfectant product guidance^22^ that “The product should demonstrate complete inactivation of the virus at all dilutions. If cytotoxicity is present, the virus control titer should be increased to demonstrate a ≥ 3 log_10_ reduction in viral titer beyond the cytotoxic level.” For disinfectants that are non-cytotoxic to the cellular infectivity assays used for demonstrating efficacy, a 4-log_10_ reduction in viral titer is typically considered to be effective. However, as we have previously done when dealing with especially lethal viruses, such as Ebola virus^23,24^, we extended the stringency of the assay for detecting residual infectious virus post-exposure to the microbicides by conducting the plate safety test. The latter enabled any infectious virus remaining post-exposure/neutralization to amplify in Vero cells in a six-well plate format, with up to two passages onto fresh cells performed for negative wells. This additional test was used to confirm that conditions scored negative in the TCID_50_ titration assay were, in fact, free of infectious virus.

In a recently published preprint^25^, Shaffer et al. have reported on the persistence of LASV Josiah and Sauerwald isolates on hard surfaces and in water. Approximately 1.9 log_10_ reduction in titer per day was observed on high-density polyethylene (HDPE) and stainless steel surfaces for the Josiah isolate, and approximately 1.2 log_10_ per day on these surfaces for the Sauerwald isolate. These data indicate that surface contamination with infectious LASV could persist for days, depending on the initial titer of the deposited virus. Decay rates for the two isolates in deionized water (0.1 to 0.15 log_10_ per day) and wastewater (0.6 to 0.8 log_10_ per day) were observed^25^. Inactivation of the two LASV isolates by sodium hypochlorite (1, 5, or 10 mg/L [ppm]) was concentration dependent, with the Sauerwald isolate displaying greater susceptibility to inactivation, with no reasons for this difference being offered in the paper^25^. Greater than 4 log_10_ inactivation of LASV occurred within 5 min contact time with 1 to 10 mg/L [ppm] sodium hypochlorite for each isolate^25^. These sodium hypochlorite concentrations are quite low, compared to the concentration used in our study (0.5% [5000 ppm]), and to concentrations proposed previously for use against LASV (0.5–1%)^26^.

In conclusion, we have provided empirical evidence of the virucidal efficacy of commonly employed microbicidal actives (ethanol and sodium hypochlorite) and formulations of microbicidal actives (AHP, PCMX, and dual QAC) for LASV. Each of these, at the appropriate concentration and contact time, was capable of reducing the titer of infectious virus by > 6 log_10_, even in the presence of a tripartite organic load^7,27^. In future studies, we plan to explore the more stringent virucidal efficacy of a similar set of microbicidal actives and formulations against LASV in carrier-inactivation studies of the virus dried on a hard surface and performed in accordance with ASTM-E2197-11^7^.

### Supplementary Information

Supplementary Information.

## Data Availability

Supplementary information accompanies this paper at 10.1038/s41598-023-38954-5. Additional datasets used and/or analyzed during the current study are available from the corresponding author on reasonable request.
