# Supplementary material for: Efficacy of microbicidal actives and formulations for inactivation of Lassa virus in suspension
Source: Sci Rep. 2023 Aug 10;13:12983. doi: 10.1038/s41598-023-38954-5 (PMC10415271; doi:10.1038/s41598-023-38954-5)
Supplement: Supplementary file 1 — Supplementary Information. [file 41598_2023_38954_MOESM1_ESM.docx]

**Efficacy of microbicidal actives and formulations for inactivation of Lassa virus in suspension**

**Todd A. Cutts, Raymond W. Nims, Joseph R. Rubino, Julie McKinny, Jens Kuhn & M. Khalid Ijaz**

1. **Assessment of Microbicide Neutralization by Chemical Reagents**

A neutralization assay was performed to evaluate the ability of candidate neutralizing reagents to neutralize the virucidal effects of microbicides at the tested concentrations. For this study, only virus culture medium (VCM: Minimal Essential Medium containing 2% fetal calf serum and 10 units per mL penicillin/streptomycin) was assayed for neutralizing ability. LASV was diluted to approximately 10^4^ to 10^5^ TCID_50_ per mL, with 10 µL virus evaluated per control in replicates of three. The candidate reagents were evaluated for neutralization efficacy (by scoring wells containing Vero cells for CPE and GFP), and cytotoxicity to the cells (assessed using microscopic evaluation of wells for morphology and confluency of Vero cells) using the following conditions:

**Negative Control.** Cells were cultured in VCM and used as a baseline of comparison for evaluation of cytotoxicity or CPE/GFP.

**Neutralizer Control.** The candidate neutralizers to be evaluated were diluted in VCM using a 10-fold serial scheme from 10^0^ (undiluted) to 10^-3^. Neutralizer dilutions were added (50 µL) to Vero cells (n =5 replicates per dilution). Cells were scored for cytotoxicity 14 days post-inoculation.

**Positive Control.** The virus control was prepared by adding 10 µL of LASV in a tripartite soil load (10^2^ to 10^4^ TCID_50_ virus; 0.25% bovine serum albumin, 0.35% tryptone, 0.08% bovine mucin) to 990 µL of VCM. Final concentrations in the positive control were: virus (10^2^ to 10^4^ TCID_50_/mL), bovine serum albumin (BSA, 0.0025%), tryptone (0.0035%), and mucin (0.0008%). The positive control was diluted in VCM using a ten-fold dilution scheme from 10^0^ (undiluted) to 10^-3^, and 50 µL of the resulting solutions were added to Vero cells. Cells were scored for CPE/GFP 5 days post-inoculation.

**Neutralizer + Virus Controls.** To account for the effect of the neutralizer acting on the virus, neutralizer + virus controls were prepared. These were prepared in the same manner as the positive virus control, except that the 10 µL of LASV in tripartite soil load was added to 990 µL of candidate neutralizer instead of VCM. The neutralizer + virus controls were diluted in VCM using a ten-fold dilution scheme from 10^0^ (undiluted) to 10^-3^, and 50 µL of the resulting solutions were added to Vero cells. Cells were scored for cytotoxicity and CPE/GFP 5 days post-inoculation.

**Neutralizer + Disinfectant + Virus Controls.** To demonstrate the efficacy of the candidates for neutralizing viral inactivation by the microbicides, 50 µL of diluted microbicide were added to 940 µL of the candidate neutralizers. Shortly thereafter, 10 µL of LASV in tripartite soil load (concentrations of components are given above) were added and the resulting mixtures were incubated for 10 min at room temperature. The neutralizer + disinfectant + virus controls were diluted in VCM using a ten-fold dilution scheme from 10^0^ (undiluted) to 10^-3^, and 50 µL of the resulting solutions were added to Vero cells. Cells were scored for cytotoxicity and CPE/GFP 5 days post-inoculation.

**Figure S1**. Evaluation of neutralizing efficacy of VCM for 70% ethanol. Abbreviations used: N, negative control; N+D, negative + microbicide; Pos, LASV in tripartite load, no microbicide; N+V, neutralizer plus LASV; N+D+V, neutralizer plus microbicide + LASV.

**Figure S2**. Evaluation of neutralizing efficacy of VCM for 0.5% sodium hypochlorite. Abbreviations used: N, negative control; N+D, negative + microbicide; Pos, LASV in tripartite load, no microbicide; N+V, neutralizer plus LASV; N+D+V, neutralizer plus microbicide + LASV.

1. **Assessment of Microbicide Neutralization by Chemical Reagents and using Amicon Columns.**

Neutralizing reagents or mechanical removal using Amicon filter columns were evaluated for ability to neutralize the virucidal effects of the microbicide formulations (Dual QAC, AHP, and PCMX) to allow investigation of specific contact times, and/or to mitigate the cytotoxic effects of the microbicides on the Vero cells used to assay residual infectious virus. The ability to successfully recover virus from the filtration process was also evaluated. The procedures used are described below.

To determine the potential net loss of virus incurred through use of an Amicon 30 kDa YM30 filtration column, prepared LASV stock was diluted in 5 mL of VCM to a titer of 2.5 log_10_ TCID_50_/mL. Three 500-μL aliquots were removed from the stock and tittered, to act a positive inoculum control. The remaining inoculum was added in 500-μL aliquots to six YM30 columns and prepared per the manufacturer’s protocol. After one spin and wash cycle, three tubes were removed and the virus from each was eluted and tittered, to serve as a measure of virus loss after one wash. The remaining three tubes underwent an additional 2 washes. The virus was eluted and tittered, to assess the possible loss of virus after 3 wash cycles. No significant loss of virus was observed between the positive control and the one or three wash cycles. The results are presented below.

**Figure S3**. Impact of use of Amicon YM30 columns on LASV titer.

1. **Assessment of Microbicide Removal by Amicon YM30 columns**

A neutralization assay was performed to evaluate the ability of Amicon YM30 columns to neutralize the virucidal effects of formulated microbicides at the tested concentrations. For this study, only VCM was used to chemically neutralize the formulated microbicides prior to being filtered through the YM30 column. LASV was diluted to approximately 10^6^ to 10^7^ TCID_50_ per mL, with 10 µL virus evaluated per control in replicates of three. The ability of the VCM plus YM30 column to neutralize the formulated microbicides was evaluated by scoring wells of Vero cells exposed to the post-neutralization samples for CPE and GFP. Potential cytotoxicity to Vero cells exposed to the post-neutralization samples was assessed by microscopic evaluation of wells for morphology and confluency of Vero cells using the following conditions:

**Negative Control.** Cells were cultured in VCM and used as a baseline of comparison for evaluation of cytotoxicity or CPE/GFP.

**Neutralizer + Disinfectant Control.** Residual microbicide remaining in the column could adversely affect the viability of the cells. The formulated microbicides were prepared with 50 μL being added to 500 μL of VCM. The mixture was then spun though Amicon YM30 columns following the manufacturer’s protocol. Formulated PCMX and AHP required only 1 spin and wash to remove residual virucidal activity, however four spins and washes were required to completely neutralize the virucidal effects of the Dual QAC. Retentates were eluted and diluted in 500 μL VCM and 10-fold diluted from 10^0^ (undiluted) to 10^-3^. Neutralizer dilutions were added (50 µL) to Vero cells (n = 5 replicates per dilution), and the cells were scored for cytotoxicity 5 days post-inoculation.

**Positive Control.** The virus control was prepared by adding 10 µL of LASV in a tripartite soil load (10^3^ to 10^7^ TCID_50_ virus; 0.25% bovine serum albumin, 0.35% tryptone, 0.08% bovine mucin) to 990 µL of VCM. Final concentrations in the positive control were: virus (10^3^ to 10^7^ TCID_50_/mL), BSA (0.0025%), tryptone (0.0035%), and mucin (0.0008%). The positive control was diluted in VCM using a ten-fold dilution scheme from 10^0^ (undiluted) to 10^-7^, and 50 µL of the resulting solutions were added to Vero cells. Cells were scored for CPE/GFP 5 days post-inoculation and the titer of the positive control in TCID_50_ per mL was determined.

**Neutralizer + Virus Controls.** To account for the effect of the Amicon YM30 column acting on the virus, a neutralizer + virus control was prepared. 10 µL of LASV in tripartite soil load was added to 500 µL of VCM and centrifuged through the Amicon column using the manufacturer’s protocol. The prepared virus was eluted from the column and diluted in VCM using a ten-fold dilution scheme from 10^0^ (undiluted) to 10^-7^, and 50 µL of the resulting solutions were added to Vero cells. Cells were scored for CPE/GFP 5 days post-inoculation and the titer of the diluted neutralization mixture in TCID_50_ per mL was determined.

**Neutralizer + Disinfectant + Virus Controls.** To assess the ability of the Amicon YM30 column to neutralize the virucidal activity of the formulated microbicides, a neutralizer + disinfectant + virus control condition was prepared. The formulated microbicides were prepared, with 50 µL microbicide added to 500 µL of VCM. The mixtures were then spun though Amicon YM30 columns using the manufacturer’s protocol. Formulated PCMX and AHP required only 1 spin and wash to neutralize the virucidal activity of the microbicides, however four spins and washes were required to neutralize the virucidal activity of the Dual QAC. Retentates were eluted and diluted in 500 µL VCM, with 10 µL of LASV in tripartite soil load being added. Samples were diluted in VCM using a ten-fold dilution scheme from 10^0^ (undiluted) to 10^-7^, and 50 µL of the resulting solutions were added to Vero cells. Cells were scored for CPE/GFP 5 days post-inoculation and the titer of the diluted eluate in TCID_50_ per mL was determined.

**Figure S4**. Evaluation of neutralizing efficacy of VCM plus Amicon column for formulated PCMX (1:40 dilution). Abbreviations used: N, negative control; N+D, negative + microbicide; Pos, LASV in tripartite load, no microbicide; N+V, neutralizer plus LASV; N+D+V, neutralizer plus microbicide + LASV.

**Figure S5**. Evaluation of neutralizing efficacy of VCM plus Amicon column for formulated AHP. Abbreviations used: N, negative control; N+D, negative + microbicide; Pos, LASV in tripartite load, no microbicide; N+V, neutralizer plus LASV; N+D+V, neutralizer plus microbicide + LASV.

**Results of Neutralization Effectiveness Evaluation**. It was found that 0.5% sodium hypochlorite and 67% ethanol could be adequately neutralized using VCM (MEM + 2% FCS +10 units/mL penicillin/streptomycin) alone. As mentioned above, neutralization of AHP, PCMX, and dual QAC formulations using VCM and Amicon columns was required.
